# Pylorus‐preserving versus Pylorus‐resecting: Impact on dynamic changes of nutrition and body composition in pancreatic cancer patients before and after pancreatoduodenectomy

**DOI:** 10.1002/cam4.5155

**Published:** 2022-08-26

**Authors:** Qianna Jin, Qianqian Ren, Xiaona Chang, Xiaoming Lu, Guobin Wang, Nan He

**Affiliations:** ^1^ Department of Radiology, Union Hospital, Tongji Medical College Huazhong University of Science and Technology Wuhan China; ^2^ Hubei Province Key Laboratory of Molecular Imaging Wuhan China; ^3^ Department of Pathology, Union Hospital, Tongji Medical College Huazhong University of Science and Technology Wuhan China; ^4^ Cancer Center, Union Hospital, Tongji Medical College Huazhong University of Science and Technology Wuhan China; ^5^ Department of Gastrointestinal Surgery, Union Hospital, Tongji Medical College Huazhong University of Science and Technology Wuhan China

**Keywords:** body composition, CT, pancreatoduodenectomy, pylorus‐resecting pancreatoduodenectomy, sarcopenia

## Abstract

**Objectives:**

To investigate if different methods of pancreatoduodenectomy (with or without pyloric preservation) would have different impacts on postoperative nutrition and body composition changes among pancreatic cancer patients.

**Methods:**

Demographic and clinicopathological data, perioperative data were collected, body composition (e.g. skeletal muscle cross‐sectional area [CSA], visceral fat area [VFA]) were evaluated with abdominal CT before and after surgery. Sarcopenia patients' proportion changes were also recorded.

**Results:**

The hospital stay in the PRPD group was significantly less than that in the PPPD group (*p* < 0.05). A significant difference was found in CSA, skeletal muscle index (SMI), VFA, VFA/CSA and albumin (ALB) in both groups between preoperative, 3, and 12 months after surgery. The loss of visceral fat in the PRPD group was more prominent than that in the PPPD group at 3 months and 12 months after surgery (*p* < 0.05). VFA/CSA was higher in the PPPD group than in the PRPD group (3 months: *p* < 0.05, 12 months: *p* < 0.001). The proportion of sarcopenic patients increased significantly over time in the PPPD and PRPD groups (*p* < 0.001).

**Conclusions:**

Postoperative CSA and VFA continued to significantly decrease in both PPPD and PRPD groups, while the incidence of sarcopenia continued to increase. Compared with PRPD, PPPD has a protective effect on visceral fat. PPPD may contribute to better maintaining visceral fat mass and blood ALB levels. CT quantification can be an objective and effective method to evaluate the nutritional status of pancreatic cancer patients during the pre‐ and postoperative period and can provide a useful objective basis for guiding clinical treatment.

## Graphical abstract

CT quantitatively analyzed the changes in skeletal muscle and visceral fat in patients with pancreatic cancer before and after surgery, to dynamically and objectively reflect the impact of different surgical methods on the pre‐ and postoperative nutritional status of patients.

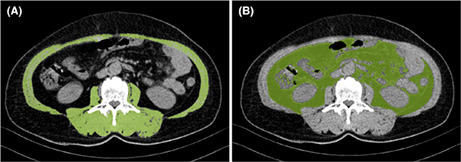

## 1 INTRODUCTION

Pancreatic cancer is one of the most fatal malignant tumors and is the fourth most common cause of death due to cancer worldwide. Mortality is always high in developed countries, in recent years, China has a similar disease spectrum to developed countries due to lifestyle changes such as consumption of high‐calorie foods, smoking, and reduced physical activity.^1^ Pancreatic cancer ranks high in morbidity and mortality among malignant tumors in China.^2^ Pancreaticoduodenectomy (PD) offers the only curative option for pancreatic cancer patients with resectable lesions. Pylorus‐preserving pancreatoduodenectomy (PPPD) and pylorus‐resecting pancreatoduodenectomy (PRPD) are two widely used stomach‐sparing modifications of the classic Whipple procedure.^3^, ^4^, ^5^ As the long‐term survival for patients who performed PD is poor, while functional and nutritional impairments are common, the postoperative quality of life is paramount.^6^, ^7^ Nutritional disorders significantly impair the quality of life for patients after PD. Many patients undergoing PD have experienced weight loss due to chronic malnutrition, cancer cachexia syndrome, malabsorption, or dyspepsia.^8^, ^9^, ^10^, ^11^ It is especially important to assess the changes in body composition after PD, and the prognosis is closely related to the nutritional status of each patient, which is crucial for the postoperative quality of life of the patient.^7^ Although some studies have suggested that preoperative sarcopenia may indicate a worse prognosis in pancreatic cancer patients undergoing surgery,^12^, ^13^ no study has investigated if different methods of PD would have different impacts on postoperative nutrition and body composition changes (such as abdominal muscles, visceral fat) among pancreatic cancer patients. Computed tomography (CT) is a routine follow‐up examination of pancreatic cancer patients and one of the most advanced imaging techniques available today, providing a highly accurate estimate of body composition.^14^, ^15^ The semi‐automatic method can quickly calculate the area of the visceral fat and abdominal muscle, with good repeatability, and it is easy to obtain during the postoperative period. Therefore, we used the data from CT scans to analyze changes in pancreatic cancer patients' body composition before and after surgery and to investigate whether PD with or without pylorus preservation would have an influence on body composition and nutritional status changes in postoperative patients.

## 2 MATERIALS & METHODS

### 2.1 Inclusion and exclusion criteria

We retrospectively analyzed medical records of all adult patients (age > 18 years) who underwent pancreatectomy at Wuhan Union Hospital between January 1, 2013 and May 31, 2019. Patients with pancreatic cancer were included in this study if they had undergone PPPD or PRPD, with available preoperative and postoperative CT scans. Both PPPD and PRPD procedures were suitable for patients with ductal adenocarcinoma, cholangiocarcinoma, Vater papillary carcinoma, and duodenal carcinoma, as long as the pylorus and stomach were not involved. In our institution, which procedure to choose depends on the surgical habits of different surgeons. Patients with a follow‐up fewer than 12 months, other concurrent surgeries, and other preoperative treatments (e.g., chemotherapy) were excluded. A total of 298 patients were involved in this study.

### 2.2 Patient characteristics

Patient demographics, including age, gender, body mass index (BMI), height, weight, comorbidities (e.g. cardiovascular or hepatic disease, or diabetes), and American Society of Anesthesiologists (ASA) grade were recorded at first admission. Laboratory tests such as total lymphocyte count (TLC) and albumin were checked before surgery and during follow‐up. Type of operation (PPPD or PRPD) was recorded, pathological data as tumor size, histology type, node and perineural infiltration were recorded as well. Perioperative data and hospital course were also collected, including operating time, blood loss, postoperative stay, delayed gastric emptying (DGE), performed relaparotomy or not, and complication morbidity. Data on whether patients received postoperative chemotherapy were also recorded.

### 2.3 CT quantification to assess body composition

**FIGURE 1:**
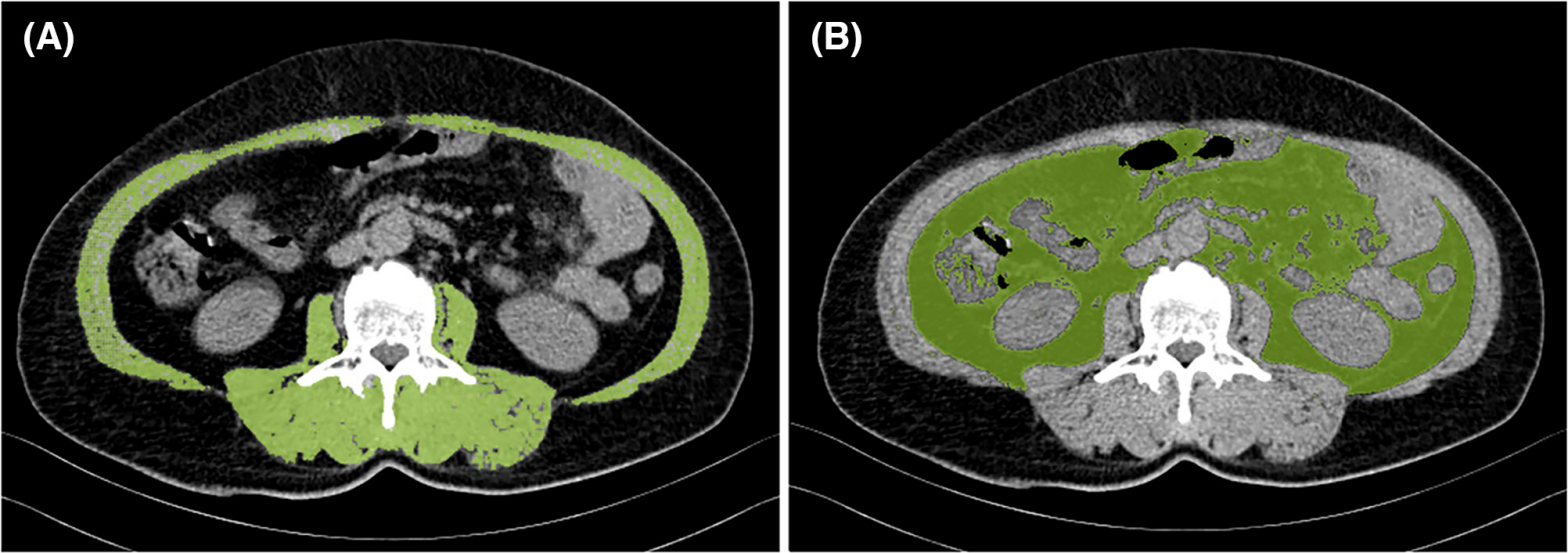
(A) The cross‐sectional area (CSA) of the skeletal muscle, and (B) the visceral fat area (VFA) at the level of the third lumbar vertebrae

Data obtained from abdominal CT scans were entered into a semi‐automatic volume tool (Syngo Volume tool, Siemens Healthcare) to assess body composition. The patient's baseline abdominal CT (CT0) examination time was within 30 days before surgery (0–30 days, mean 7 days), and the follow‐up CT examinations were included as CT1 (short‐term): CT examination 3 months after surgery (83–96 days, mean 90.2 days), CT2 (long‐term): CT examination 1 year after operation (346–385 days, mean 360 days). Abdominal cross‐sectional area (CSA) (cm^2^) was measured at the level of the third lumbar vertebra, including all muscles bilaterally in this area (internal/external obliques, quadratus lumborum, transversus abdominis, paraspinous, psoas, and rectus abdominis). Muscle density was corrected by using Hounsfield Units (HU) range −29 to 150, visceral fat area (VFA) (cm^2^) was delineated and obtained on CT by using the adipose tissue threshold (−190 to −130HU) (Figure 1). CSA was adjusted to Skeletal Muscle Index (SMI) by dividing the muscle area by the square of the patient's height, SMI = CSA (cm^2^)/Height (m^2^). Sarcopenia was defined: <52.4 cm^2^/m^2^ in men and <38.4 cm^2^/m^2^ in women.^15^ All data were collected in a retrospective manner after receiving appropriate approval by the Institutional Review Board.

### 2.4 Statistical analysis

Quantitative data for statistical variables were expressed as means ± standard deviation (SD). Variables comparisons were performed by the chi‐square test or t‐test. Wilcoxon signed‐rank test was used to identify significant differences of mean values before and after operation. Mann–Whitney‐U‐test was used to identify significant differences of mean values in PPPD and PRPD group. Significance was accepted at the probability level of 0.05. All statistical analyzes were performed using SPSS software version 17.0 (SPSS Inc.).

## 3 RESULTS

### 3.1 Patients characters

Comparing the data between the PPPD and PRPD groups (including demographic, clinicopathological data, perioperative data, and hospital course), there was no significant difference in gender, age, body weight, BMI, comorbidity (ex. cardiovascular or hepatic disease, diabetes), histology type, tumor size, positive lymph, and perineural infiltration. The postoperative hospital stay and the incidence of delayed gastric emptying were significantly lower in the PRPD group than those in the PPPD group (*p* = 0.00519, *p* = 0.032, respectively). No significant differences were found in operating time, blood loss, relaparotomy, and morbidity between PPPD and PRPD groups. The majority of the patients performed postoperative chemotherapy, no significant difference was found between PPPD and PRPD groups. All of the above results were shown in Table 1.

**TABLE 1:** Patients characters

Demographic and clinicopathological data	PPPD (131)	PRPD (167)	*p*
Age, years (mean ± SD)	61.87 ± 9.01	62.35 ± 9.22	0.65
Sex			0.38
Male	97	116	
Female	34	51	
Body weight (kg)	66.2 ± 15.5	65.7 ± 16.1	0.778
BMI	23.9 ± 4.8	23.8 ± 4.6	0.941
Comorbidity			0.4922
Cardiovascular	26	37	
Diabetes	8	11	
Hepatic	2	4	
ASA grade			0.4233
ASA I + II	79	93	
ASA III + IV	52	74	
Tumor size (cm) (mean ± SD)	2.69 ± 1.1	2.67 ± 1.2	0.893728
Histology			
Ductal adenocarcinoma	88	111	0.8974
Carcinoma papilla	25	38	0.4412
Carcinoma distal bile duct	16	17	0.5786
Duodenal cancer	2	1	0.4258
Nodal positive	83	101	0.6117
Perineural infiltration	30(+)/101	36(+)/131	0.7816
Perioperative data and hospital course			
Operating time, min (mean ± SD)	359 ± 44	350 ± 46	0.068
Blood loss (ml)	402 ± 77	405 ± 78	0.849
Postoperative stay (days), (mean ± SD)	18.4 ± 8.3	15.9 ± 6.8	**0.005199**
Delayed gastric emptying (DGE)	26 (19.8%)	18 (10.8%)	**0.0328**
Relaparotomy	16 (12%)	19 (11%)	0.8238
Complication morbidity			0.6243
Pulmonary	2	1	
Leakage hepaticojejunostomy	2	2	
Leckage Pancreaticojejunostomy	8	13	
Intra‐abdominal abscess	0	1	
Pancreatitis	2	1	
Wound infection	1	1	
Bleeding	9	8	
Postoperative chemotherapy			0.493
Yes	103	125	
No	28	42	

*Note*: Significance of bold means *p* value <0.05.

Abbreviation: SD, standard deviation.

### 3.2 Changes on nutrition and body composition in PPPD & PRPD patients before and after operation

In the PPPD group, a continuous decrease was found in CSA, SMI, VFA, and VFA/CSA, and TLC. Patients' ALB slightly decreased in the 3rd month compared with the preoperative state, with a minimal increase during 3 to 12 months. A significant difference was found in CSA, SMI, VFA, VFA/CSA ALB, and TLC between preoperative and 3 months after surgery, preoperative and 12 months after surgery, 3 months and 12 months after surgery.

In the PRPD group, a continuous decrease was found in CSA, SMI, VFA, VFA/CSA. ALB slightly decreased in the 3rd month compared with preoperative, with a minimal increase during 3 to 12 months. A significant difference was found in CSA, SMI, VFA, VFA/CSA, and ALB in the PRPD group between preoperative and 3 months after surgery, preoperative and 12 months after surgery, 3 months and 12 months after surgery. TLC in the PRPD group increased in the 3rd month after surgery, and decreased close to the preoperative level in the 12th month after surgery. A significant difference was found in TLC between preoperative and 3 months after surgery, 3 months and 12 months after surgery, and no significant difference was found between 12 months after surgery and preoperative.

Our results showed that in PPPD and PRPD groups, both muscle tissue (CSA, SMI), adipose tissue (VFA), and VFA/CSA ratio significantly decreased after surgery within a short period of time (3 months). VFA reduction was very obvious (−36.48 in PPPD and −42.29 in PRPD). In both the PPPD group and PRPD group, albumin decreased significantly in the first 3 months after the operation and increased slightly in the 12th month after the surgery, but it was still lower than the preoperative state. (Table 2).

**TABLE 2:** Body composition and nutrition in PPPD & PRPD patients before and after operation, Wilcoxon‐signed‐rank‐test for identifying significant differences of mean values before and after operation

	CSA	SMI	VFA	VFA/CSA	ALB	TLC
PPPD (*n* = 131)						
preOP						
Mean	131.38	47.56	117.1	0.92	4.01	2.26
SD	28.27	9.27	22.9	0.25	0.45	1.55
3 months postOP						
Mean	124.72	45.17	80.62	0.64	3.81	2.23
SD	27.32	8.99	23.37	0.12	0.51	0.78
12 months postOP						
Mean	117.5	42.54	51.18	0.43	3.85	2.17
SD	27.53	9.09	19.47	0.12	0.56	0.85
Difference between 3 months postOP and preOP						
Mean	−6.66	−2.39	−36.48	−0.28	−0.2	−0.03
*p* value	**<0.001**	**<0.001**	**<0.001**	**<0.001**	**<0.001**	**<0.001**
Difference between 12 months postOP and preOP						
Mean	−13.88	−5.02	−65.92	−0.49	−0.16	−0.09
*p* value	**<0.001**	**<0.001**	**<0.001**	**<0.001**	**<0.001**	**<0.001**
Difference between 12 months postOP and 3 months postOP						
Mean	−7.22	−2.63	−29.44	−0.21	0.04	−0.06
*p* value	**<0.001**	**<0.001**	**<0.001**	**<0.001**	**<0.001**	**<0.001**
PRPD (*n* = 167)						
preOP						
Mean	128.93	47.11	116.3	0.93	4.04	2.08
SD	25.89	7.76	20.3	0.23	0.48	0.62
3 months postOP						
Mean	121.29	44.32	74.01	0.61	3.65	2.18
SD	24.48	7.64	21.52	0.11	0.37	0.56
12 months postOP						
Mean	115.37	42.16	45.15	0.38	3.7	2.04
SD	25.27	7.93	17.77	0.1	0.44	0.56
Difference between 3 months postOP and preOP						
Mean	−7.64	−2.79	−42.29	−0.32	−0.39	0.1
*p* value	**<0.001**	**<0.001**	**<0.001**	**<0.001**	**<0.001**	**<0.001**
Difference between 12 months postOP and preOP						
Mean	−13.56	−4.95	−71.15	−0.55	−0.34	−0.04
*p* value	**<0.001**	**<0.001**	**<0.001**	**<0.001**	**<0.001**	0.606
Difference between 12 months postOP and 3 months postOP						
Mean	−5.92	−2.16	−28.86	−0.23	0.05	−0.14
*p* value	**<0.001**	**<0.001**	**<0.001**	**<0.001**	**<0.001**	**<0.001**

*Note*: Significance of bold means *p* value <0.05.

Abbreviations: ALB, albumin; CSA, cross‐sectional area; postOP, after operation; PPPD, pylorus‐preserving pancreatoduodenectomy; preOP, before operation; PRPD, pylorus‐resecting pancreatoduodenectomy; SMI, skeletal muscle index; TLC, total lymphocyte count; VFA, visceral fat area.

### 3.3 Differences in changes on body composition and nutrition between PPPD & PRPD group

With regard to body composition, there was no significant difference in skeletal muscle (CSA, SMI) and visceral fat area (VFA) between PPPD and PRPD groups before operation. Visceral fat was found to be most affected by pancreaticoduodenectomy. The reduction of visceral fat was more prominent in the PRPD group than the PPPD group with statistically significant difference in both 3 and 12 months after surgery (*p* < 0.05). CSA and SMI showed a continuous decrease at 3 and 12 months after surgery in both PPPD and PRPD groups, although there was no significant difference between the two groups before and after surgery. VFA/CSA, an indicator of sarcopenic obesity, decreased more prominent in the PRPD group than in the PPPD group, with a significant statistical difference (3 months: *p* < 0.05, 12 months: *p* < 0.001). (Table 3).

**TABLE 3:** Differences in changes of body composition and nutrition in PPPD & PRPD patients before and after operation, Mann–Whitney‐U‐test for identifying significant differences of mean values in PPPD and PRPD group

	PPPD (*n* = 131)	PRPD (*n* = 167)	*p* value
CSA (cm^2^)			
preOP	131.38 ± 28.27	128.93 ± 25.89	0.443
3 months postOP	124.72 ± 27.32	121.29 ± 24.48	0.249
12 months postOP	117.50 ± 27.53	115.37 ± 25.27	0.493
SMI (cm^2^/m^2^)			
preOP	47.56 ± 9.27	47.11 ± 7.76	0.651
3 months postOP	45.17 ± 8.99	44.32 ± 7.64	0.388
12 months postOP	42.54 ± 9.09	42.16 ± 7.93	0.704
VFA (cm^2^)			
preOP	117.1 ± 22.9	116.3 ± 20.3	0.753
3 months postOP	80.62 ± 23.37	74.01 ± 21.52	**0.013**
12 months postOP	51.18 ± 19.47	45.15 ± 17.77	**0.006**
VFA/CSA			
preOP	0.92 ± 0.25	0.93 ± 0.23	0.816
3 months postOP	0.64 ± 0.12	0.61 ± 0.11	**0.011**
12 months postOP	0.43 ± 0.12	0.38 ± 0.10	**<0.001**
ALB (g/dL)			
preOP	4.01 ± 0.45	4.04 ± 0.48	0.640
3 months postOP	3.81 ± 0.51	3.65 ± 0.37	**0.004**
12 months postOP	3.85 ± 0.56	3.70 ± 0.44	**0.012**
TLC (10^9^/L)			
preOP	2.26 ± 1.55	2.08 ± 0.62	0.195
3 months postOP	2.23 ± 0.78	2.18 ± 0.56	0.550
12 months postOP	2.17 ± 0.85	2.04 ± 0.56	0.133

*Note*: Significance of bold means *p* value <0.05.

Abbreviations: ALB, albumin; CSA, cross‐sectional area; postOP, after operation; PPPD, pylorus‐preserving pancreatoduodenectomy; preOP, before operation; PRPD, pylorus‐resecting pancreatoduodenectomy; SMI, skeletal muscle index; TLC, total lymphocyte count; VFA, visceral fat area.

There were significant differences in albumin levels between PPPD and PRPD groups at 3 months and 12 months after surgery, but no difference was found in Albumin levels before operation between PPPD and PRPD groups. There was no significant difference in TLC between the PPPD group and the PRPD group before and after surgery. (Table 3).

### 3.4 Changes of sarcopenia patients' proportion in PRPD & PPPD patients

Referring to sarcopenia criteria,^14^ the differences in the proportion of patients with sarcopenia among PPPD and PRPD groups were not statistically significant in the preoperative or postoperative state (Table 4, P1). However, the proportion of patients with sarcopenia in the PPPD and PRPD groups was found to increase over time, and the difference was statistically significant (Table 4, P2). Within 12 months after PD, the proportion of patients with sarcopenia increased to 73% in PPPD and 68% in PRPD, respectively.

**TABLE 4:** Sarcopenia patients' proportion significant increase in PRPD&PPPD patients

	PPPD (*n* = 131)		PRPD (*n* = 167)		Total (*n* = 298)		P1
pre‐OP	48	(37%)	51	(31%)	99	(33%)	0.267
3 m‐postOP	69	(53%)	96	(57%)	165	(55%)	0.4068
12 m‐postOP	96	(73%)	113	(68%)	208	(70%)	0.2929
P2	**<0.0001**		**<0.0001**		**<0.0001**		

*Note*: Significance of bold means *p* value <0.05.

Abbreviations: postOP, after operation; PPPD, pylorus‐preserving pancreatoduodenectomy; preOP, before operation; PRPD, pylorus‐resecting pancreatoduodenectomy.

## 4 DISCUSSION

The patients' nutritional status after pancreatoduodenectomy is usually poor. Cachexia, sarcopenia, and obvious weight loss are frequently observed in patients after pancreatoduodenectomy due to malnutrition, postoperative dysfunction, metabolic chaos, chemotherapy toxicity, etc. Several studies have compared the postoperative body composition and nutritional status changes in the PPPD and PRPD groups, but they were based on non‐imaging modalities such as laboratory findings, demographic characteristics, or bioelectrical impedance analysis (BIA).^5^, ^16^, ^17^ CT or MRI is currently considered the gold standard for quantitative assessment of visceral fat and intra‐abdominal muscle.^18^, ^19^ As patients after PD will routinely undergo abdominal and pelvic CT scans to detect metastasis or recurrence, CT is the optimal tool for analyzing postoperative body composition changes. To the best of our knowledge, this study is the first to dynamically compare the changes in patients' body composition before and after pancreatic cancer surgery based on CT quantitative analysis, and to explore the effects of PPPD and PRPD on nutritional status and body composition changes based on nutrition parameters and CT examinations during 12 months after PD.

Consistent with previous studies,^7^, ^20^, ^21^ our research also found no difference between PPPD and PRPD in operating time, perioperative blood loss, relaparotomy and morbidity of complication. The incidence of delayed gastric emptying (DGE) in PPPD patients was higher than that in PRPD patients in our study, which might result in prolonged postoperative nasogastric tube placement time, and pyloric spasms caused by pyloric weaning and denervation after PPPD might be a possible cause.^22^, ^23^ Post‐operation hospital stay in PRPD was shorter than in PPPD in our study, which might be due to higher DGE incidence in the PPPD group.

Our research found that in terms of body composition, both PRPD and PPPD patients showed significant muscle and abdominal fat loss in the 3rd month after surgery compared with the preoperative state, the same phenomenon existed in the 12th month after surgery, and these above differences were statistically significant. Postoperative wasting,^24^, ^25^, ^26^ chemotherapy toxicity,^27^, ^28^ delayed gastric emptying,^29^ etc., will contribute to the loss of muscle and fat in patients after PD, which will be related to poor quality of life. A previous study used BIA to observe the changes in body composition after PD^30^ and found fatmass continuously decreased after pancreatectomy. Park et al found that although some patients' body weight recovered within 6 months after surgery, their body fat continues to decrease.^31^ Our research results were consistent with these researches.

We also compared differences in changes in body composition and hematological parameters reflecting nutritional status between the PPPD and PPRD groups. It was found that these indicators (CSA, SMI, TLC) were not different between the two groups. Although the indicators reflecting skeletal muscle changes (CSA, SMI) of the two groups decreased significantly at 3rd and 12th month after surgery, there was no significant difference between PPPD and PPRD groups. Previous studies reported that although BMI change and weight loss were not statistically different between PRPD and PPPD groups, BMI and body weight were significantly lower postoperatively compared to preoperatively in each group.^17^, ^32^, ^33^ However, these studies have not been able to show which and how much body component (muscle or fat) is lost. In our study, VFA decreased more significantly in the PRPD patients than those in the PPPD, which suggested that indicators reflecting visceral fat changes (VFA, VFA/CSA) would be a more independent predictor than indicators reflecting skeletal muscle changes (CSA, SMI) to reflect postoperative body composition changes in pancreatic cancer patients. ALB of PPPD and PRPD groups of patients dropped to the lowest point at the 3rd month after surgery, and recovered slightly at the 12th month. PPPD was better than PRPD in maintaining postoperative ALB levels in this study. Although there were significant differences in ALB levels between the two groups, the ALB levels in both groups were within the normal physiological range. If we only refer to the results of postoperative blood tests, it may not be enough to objectively reflect the true nutritional status of postoperative patients. In contrast, the addition of CT quantification analysis demonstrated superiority in quantifying body composition.

The proportion of patients with sarcopenia after PD showed a continuous increase in 3 months and 12 months. Regardless of the PPPD group or the PRPD group, the proportion of patients with sarcopenia at 3 months and 12 months after the surgery was significantly higher than that before surgery with statistical significance (*p* < 0.001, Table 4). This finding is similar to previous studies in which sarcopenia was significantly increased after surgery compared to before surgery, suggesting a poorer prognosis and shorter overall survival in cancer patients.^34^ No significant difference was found in the proportion of patients with sarcopenia between PPPD and PRPD groups regardless of before and after PD in this study. Although sarcopenia is an indicator of overall survival after pancreatic cancer surgery, a recent randomized trial found no difference in overall survival after PPPD and PRPD,^17^ our study may help explain why the two surgical procedures (PPPD and PRPD) had no effect on patients' overall survival after surgery.

The present study presents some limitations. Firstly, this is a single‐center retrospective study, further work is required validation by well‐designed, prospective, multicenter studies. Secondly, postoperative chemotherapy may affect appetite and thus nutritional status, but there was no difference in the proportion of patients who received postoperative chemotherapy in our two groups, so this effect can be ignored. Thirdly, our research subjects are only Asians, and different ethnic groups may have different results, which needs to be verified in a wider cross‐regional study. Lastly, whether inconsistency in body composition between PPPD and PRPD patients affects overall quality of life and overall survival requires further studies to verify.

## 5 CONCLUSIONS

In conclusion, this study was based on CT quantitative method to measure the body composition (abdominal muscle and VFA) of patients, so as to dynamically observe the changes in body composition in pancreatic cancer patients within 12 months after surgery. Postoperative CSA and VFA continued to significantly decrease in both PPPD and PRPD groups, while the incidence of sarcopenia continued to increase. However, compared with PRPD, PPPD can contribute to better maintaining visceral fat mass and blood ALB levels. In conclusion, CT quantification can be an objective and effective method to evaluate the nutritional status of pancreatic cancer patients during the pre‐ and postoperative period and can provide a useful objective basis for guiding the clinical treatment of patients.

## AUTHOR CONTRIBUTIONS

Qianna Jin: Data curation, Formal analysis, Investigation, Methodology, Visualization, Writing ‐original draft. Qianqian Ren: Data curation, Investigation, Methodology, Visualization. Xiaona Chang: Data curation, Investigation, Methodology. Xiaoming Lu: Data curation, Investigation, Writing ‐review & editing. Guobin Wang: Data curation, Investigation, Validation, Writing ‐review & editing. Nan He: Conceptualization, Data curation, Investigation, Methodology, Project administration, Supervision, Writing ‐review & editing.

## CONFLICT OF INTEREST

All of the authors do not report relevant conflicts of interest.

## Data Availability

The datasets used in the current study are available from the corresponding author on reasonable request.

## References

[1] Ilic M , Ilic I . Epidemiology of pancreatic cancer. World J Gastroenterol. 2016;22(44):9694‐9705. 27956793 10.3748/wjg.v22.i44.9694 PMC5124974

[2] Jia X , Du P , Wu K , et al. Pancreatic cancer mortality in China: characteristics and prediction. Pancreas. 2018;47(2):233‐237. 29303909 10.1097/MPA.0000000000000976

[3] Hanna MM , Gadde R , Allen CJ , et al. Delayed gastric emptying after pancreaticoduodenectomy. J Surg Res. 2016;202(2):380‐388. 27229113 10.1016/j.jss.2015.12.053

[4] Matsumoto I , Shinzeki M , Asari S , et al. A prospective randomized comparison between pylorus‐ and subtotal stomach‐preserving pancreatoduodenectomy on postoperative delayed gastric emptying occurrence and long‐term nutritional status. J Surg Oncol. 2014;109(7):690‐696. 24619624 10.1002/jso.23566

[5] Tran KT , Smeenk HG , van Eijck CH , et al. Pylorus preserving pancreaticoduodenectomy versus standard Whipple procedure: a prospective, randomized, multicenter analysis of 170 patients with pancreatic and periampullary tumors. Ann Surg. 2004;240(5):738‐745. 15492552 10.1097/01.sla.0000143248.71964.29 PMC1356476

[6] Filip B , Hutanu I , Musina AM , et al. Functional results following pylorus‐preserving pancreatoduodenectomy with pancreaticogastrostomy. Chirurgia (Bucur). 2018;113(3):391‐398. 29981670 10.21614/chirurgia.113.3.391

[7] Niedergethmann M , Shang E , Farag Soliman M , et al. Early and enduring nutritional and functional results of pylorus preservation vs classic Whipple procedure for pancreatic cancer. Langenbecks Arch Surg. 2006;391(3):195‐202. 16491403 10.1007/s00423-005-0015-3

[8] Loh KW , Vriens MR , Gerritsen A , et al. Unintentional weight loss is the most important indicator of malnutrition among surgical cancer patients. Neth J Med. 2012;70(8):365‐369. 23065984

[9] Aslani A , Gill AJ , Roach PJ , Allen BJ , Smith RC . Preoperative body composition is influenced by the stage of operable pancreatic adenocarcinoma but does not predict survival after Whipple's procedure. HPB (Oxford). 2010;12(5):325‐333. 20590908 10.1111/j.1477-2574.2010.00171.x PMC2951821

[10] Rasmussen HH , Irtun O , Olesen SS , Drewes AM , Holst M . Nutrition in chronic pancreatitis. World J Gastroenterol. 2013;19(42):7267‐7275. 24259957 10.3748/wjg.v19.i42.7267 PMC3831208

[11] Bachmann J , Büchler MW , Friess H , Martignoni ME . Cachexia in patients with chronic pancreatitis and pancreatic cancer: impact on survival and outcome. Nutr Cancer. 2013;65(6):827‐833. 23909726 10.1080/01635581.2013.804580

[12] Di Sebastiano KM , Yang L , Zbuk K , et al. Accelerated muscle and adipose tissue loss may predict survival in pancreatic cancer patients: the relationship with diabetes and anaemia. Br J Nutr. 2013;109(2):302‐312. 23021109 10.1017/S0007114512001067

[13] Ozola Zalite I , Zykus R , Francisco Gonzalez M , et al. Influence of cachexia and sarcopenia on survival in pancreatic ductal adenocarcinoma: a systematic review. Pancreatology. 2015;15(1):19‐24. 25524484 10.1016/j.pan.2014.11.006

[14] Prado CM , Lieffers JR , McCargar LJ , et al. Prevalence and clinical implications of sarcopenic obesity in patients with solid tumours of the respiratory and gastrointestinal tracts: a population‐based study. Lancet Oncol. 2008;9(7):629‐635. 18539529 10.1016/S1470-2045(08)70153-0

[15] Martin L , Birdsell L , Macdonald N , et al. Cancer cachexia in the age of obesity: skeletal muscle depletion is a powerful prognostic factor, independent of body mass index. J Clin Oncol. 2013;31(12):1539‐1547. 23530101 10.1200/JCO.2012.45.2722

[16] Group. VATPNCS . Perioperative total parenteral nutrition in surgical patients. N Engl J Med. 1991;325(8):525‐532. 1906987 10.1056/NEJM199108223250801

[17] Klaiber U , Probst P , Huttner FJ , et al. Randomized trial of pylorus‐preserving vs. pylorus‐resecting pancreatoduodenectomy: long‐term morbidity and quality of life. J Gastrointest Surg. 2020;24(2):341‐352. 30671796 10.1007/s11605-018-04102-y

[18] Seidell JC , Bakker CJ , van der Kooy K . Imaging techniques for measuring adipose‐tissue distribution‐‐a comparison between computed tomography and 1.5‐T magnetic resonance. Am J Clin Nutr. 1990;51(6):953‐957. 2349931 10.1093/ajcn/51.6.953

[19] Shuster A , Patlas M , Pinthus JH , Mourtzakis M . The clinical importance of visceral adiposity: a critical review of methods for visceral adipose tissue analysis. Br J Radiol. 2012;85(1009):1‐10. 21937614 10.1259/bjr/38447238 PMC3473928

[20] Klaiber U , Probst P , Büchler MW , Hackert T . Pylorus preservation pancreatectomy or not. Transl Gastroenterol Hepatol. 2017;2:100. 29264438 10.21037/tgh.2017.11.15 PMC5723730

[21] Klaiber U , Probst P , Strobel O , et al. Meta‐analysis of delayed gastric emptying after pylorus‐preserving versus pylorus‐resecting pancreatoduodenectomy. Br J Surg. 2018;105(4):339‐349. 29412453 10.1002/bjs.10771

[22] Gauvin JM , Sarmiento JM , Sarr MG . Pylorus‐preserving pancreaticoduodenectomy with complete preservation of the pyloroduodenal blood supply and innervation. Arch Surg. 2003;138(11):1261‐1263. 14609879 10.1001/archsurg.138.11.1261

[23] Ha TW , Han KH , Son DG , Kim SP , Kim DK . Analysis of loss of heterozygosity in Korean patients with keratoacanthoma. J Korean Med Sci. 2005;20(2):340‐343. 15832014 10.3346/jkms.2005.20.2.340 PMC2808619

[24] Mayanagi S , Ishikawa A , Matsui K , Matsuda S , Irino T , Nakamura R , Fukuda K , Wada N , Kawakubo H , Hijikata N , Ando M , Tsuji T , Kitagawa Y ¸. Dis Esophagus 2021, 34(9):doaa121. 33306782 10.1093/dote/doaa121

[25] Papaconstantinou D , Vretakakou K , Paspala A , et al. The impact of preoperative sarcopenia on postoperative complications following esophagectomy for esophageal neoplasia: a systematic review and meta‐analysis. Dis Esophagus. 2020;33:doaa002. 10.1093/dote/doaa002 32193528

[26] Zhang YLZ , Jiang L , Xue Z , et al. Marked loss of adipose tissue during neoadjuvant therapy as a predictor for poor prognosis in patients with gastric cancer: a retrospective cohort study. J Hum Nutr Diet. 2021;34:585‐594. 33491826 10.1111/jhn.12861

[27] Takeda T , Sasaki T , Mie T , et al. The impact of body composition on short‐term outcomes of neoadjuvant chemotherapy with gemcitabine plus S‐1 in patients with resectable pancreatic cancer. Jpn J Clin Oncol. 2021;51:604‐611. 33479765 10.1093/jjco/hyaa247

[28] Amrute‐Nayak M , Pegoli G , Holler T , Lopez‐Davila AJ , Lanzuolo C , Nayak A . Chemotherapy triggers cachexia by deregulating synergetic function of histone‐modifying enzymes. J Cachexia Sarcopenia Muscle. 2020;12:159‐176. 33305533 10.1002/jcsm.12645 PMC7890149

[29] Balzano G , Zerbi A , Braga M , Rocchetti S , Beneduce AA , Di Carlo V . Fast‐track recovery programme after pancreatico‐ duodenectomy reduces delayed gastric emptying. Br J Surg. 2008;95(11):1387‐1393. 18844251 10.1002/bjs.6324

[30] Aslani A , Roach PJ , Smith RC . Long‐term changes in body composition after pancreaticoduodenectomy. ANZ J Surg. 2012;82(3):173‐178. 22510129 10.1111/j.1445-2197.2011.05970.x

[31] Park JW , Jang JY , Kim EJ , et al. Effects of pancreatectomy on nutritional state, pancreatic function and quality of life. Br J Surg. 2013;100(8):1064‐1070. 23616030 10.1002/bjs.9146

[32] Imamura N , Chijiiwa K , Ohuchida J , et al. Prospective randomized clinical trial of a change in gastric emptying and nutritional status after a pylorus‐ preserving pancreaticoduodenectomy: comparison between an antecolic and a vertical retrocolic duodenojejunostomy. HPB. 2014;16(4):384‐394. 23991719 10.1111/hpb.12153 PMC3967891

[33] Kawai M , Tani M , Hirono S , Okada K , Miyazawa M , Yamaue H . Pylorus‐resecting pancreaticoduodenectomy offers long‐term outcomes similar to those of pylorus‐preserving pancreaticoduodenectomy: results of a prospective study. World J Surg. 2014;38(6):1476‐1483. 24370543 10.1007/s00268-013-2420-z

[34] Trejo‐Avila M , Bozada‐Gutierrez K , Valenzuela‐Salazar C , Herrera‐Esquivel J , Moreno‐Portillo M . Sarcopenia predicts worse postoperative outcomes and decreased survival rates in patients with colorectal cancer: a systematic review and meta‐analysis. Int J Colorectal Dis. 2021;36:1077‐1096. 33481108 10.1007/s00384-021-03839-4

